# Rare Copy Number Variants Contribute to Congenital Left-Sided Heart Disease

**DOI:** 10.1371/journal.pgen.1002903

**Published:** 2012-09-06

**Authors:** Marc-Phillip Hitz, Louis-Philippe Lemieux-Perreault, Christian Marshall, Yassamin Feroz-Zada, Robbie Davies, Shi Wei Yang, Anath Christopher Lionel, Guylaine D'Amours, Emmanuelle Lemyre, Rebecca Cullum, Jean-Luc Bigras, Maryse Thibeault, Philippe Chetaille, Alexandre Montpetit, Paul Khairy, Bert Overduin, Sabine Klaassen, Pamela Hoodless, Mona Nemer, Alexandre F. R. Stewart, Cornelius Boerkoel, Stephen W. Scherer, Andrea Richter, Marie-Pierre Dubé, Gregor Andelfinger

**Affiliations:** 1 Cardiovascular Genetics, Department of Pediatrics, Centre Hospitalier Universitaire Sainte Justine, Université de Montréal, Montréal, Québec, Canada; 2 Wellcome Trust Sanger Institute, Wellcome Trust Genome Campus, Hinxton, United Kingdom; 3 Adult Congenital Heart Centre, Montreal Heart Institute, Université de Montréal, Montréal, Québec, Canada; 4 The Centre for Applied Genomics and Program in Genetics and Genome Biology, The Hospital for Sick Children, Toronto, Ontario, Canada; 5 University of Ottawa Heart Institute, Ottawa, Ontario, Canada; 6 Service of Medical Genetics, Department of Pediatrics, Centre Hospitalier Universitaire Sainte Justine, Université de Montréal, Montréal, Québec, Canada; 7 Terry Fox Laboratory, British Columbia Cancer Agency, Vancouver, British Columbia, Canada; 8 Cardiology Service, Centre Mère-Enfants, Centre Hospitalier Universitaire de Québec, Université de Laval, Québec City, Québec, Canada; 9 Genome Quebec Innovation Centre, McGill University, Montréal, Québec, Canada; 10 European Molecular Biology Laboratory–European Bioinformatics Institute, Wellcome Trust Genome Campus, Hinxton, United Kingdom; 11 Experimental and Clinical Research Center, Max-Delbrück-Center for Molecular Medicine, Berlin, Germany; 12 Department of Biochemistry, Microbiology, and Immunology, University of Ottawa, Ottawa, Ontario, Canada; 13 Ruddy Canadian Cardiovascular Genetics Centre, University of Ottawa Heart Institute, Ottawa, Ontario, Canada; 14 Child and Family Research Institute, Department of Medical Genetics, University of British Columbia, Vancouver, British Columbia, Canada; University of Pennsylvania, United States of America

## Abstract

Left-sided congenital heart disease (CHD) encompasses a spectrum of malformations that range from bicuspid aortic valve to hypoplastic left heart syndrome. It contributes significantly to infant mortality and has serious implications in adult cardiology. Although left-sided CHD is known to be highly heritable, the underlying genetic determinants are largely unidentified. In this study, we sought to determine the impact of structural genomic variation on left-sided CHD and compared multiplex families (464 individuals with 174 affecteds (37.5%) in 59 multiplex families and 8 trios) to 1,582 well-phenotyped controls. 73 unique inherited or de novo CNVs in 54 individuals were identified in the left-sided CHD cohort. After stringent filtering, our gene inventory reveals 25 new candidates for LS-CHD pathogenesis, such as *SMC1A*, *MFAP4*, and *CTHRC1*, and overlaps with several known syndromic loci. Conservative estimation examining the overlap of the prioritized gene content with CNVs present only in affected individuals in our cohort implies a strong effect for unique CNVs in at least 10% of left-sided CHD cases. Enrichment testing of gene content in all identified CNVs showed a significant association with angiogenesis. In this first family-based CNV study of left-sided CHD, we found that both co-segregating and *de novo* events associate with disease in a complex fashion at structural genomic level. Often viewed as an anatomically circumscript disease, a subset of left-sided CHD may in fact reflect more general genetic perturbations of angiogenesis and/or vascular biology.

## Author Summary

Congenital heart disease (CHD) is the leading malformation among all newborns, and one of the leading causes of morbidity and mortality in Western countries. Left-sided CHD (LS-CHD) encompasses a spectrum ranging from bicuspid aortic valve to aortic stenosis and hypoplastic left heart syndrome with familial clustering. To date, the genetic causes for LS-CHD remain unknown in the majority of patients. To determine the impact of structural genomic variation in multiplex families with LS-CHD, we searched for unique or rare copy number variants present only in affected members of a multiplex family cohort (N total = 464, N affected members = 174 (37.5%)) and absent from 1,582 controls free from LS-CHD. A stringent filter based on *in silico* prioritization and gene expression analysis during development allowed us to identify genes associated with LS-CHD. Our study revealed 25 new candidate genes for LS-CHD, such as *SMC1A*, *MFAP4*, and *CTHRC1*, and overlap with known syndromic loci. We estimate that unique copy number variants contribute to at least 10% of left-sided CHD cases, with a gene content suggesting broader perturbations of angiogenesis at the base of LS-CHD.

## Introduction

Left-sided congenital heart disease (LS-CHD) is one of the most prevalent and severe cardiac malformations. The spectrum includes bicuspid aortic valve (BAV), aortic valve stenosis (AS), coarctation of the aorta (CoA) and hypoplastic left heart syndrome (HLHS). Several observations, such as familial clustering as well as statistical evidence from heritability analyses, suggest that LS-CHD is strongly determined by genetic factors [1]–[3]. Linkage analyses have revealed several significant loci in BAV, HLHS and other forms of LS-CHD, as well as interrelatedness of subsets of BAV and HLHS [4]–[6]. In human and mouse models, mutations in key cardiac regulators (*e.g*, *NOTCH1, NKX2–5, GATA5*) can cause LS-CHD [7]–[9]. Genotype-phenotype correlations have been established for syndromic conditions, often with highly variable expressivity including LS-CHD as a feature, such as de Lange, Holt-Oram and Jacobsen syndromes [10], [11]. Recently, structural genomic variants have been implicated in the pathogenesis of congenital heart disease, but the extent to which copy number variants (CNVs) contribute to LS-CHD and its heritability has not yet been examined in detail [12], [13]. We sought to further delineate the role of such variants and hypothesized that multiplex families enriched in LS-CHD phenotypes exhibit rare, causative CNVs not detectable by linkage analysis. The cohort was assembled almost exclusively within the French-Canadian population, which is characterized by a marked founder effect and has previously led to the identification of numerous disease genes [14]. We surveyed 464 genomes/individuals in 59 multiplex families and 8 trios of a pedigree-based LS-CHD cohort from Quebec using the Affymetrix Genome-Wide Human SNP Array 6.0 array [15]. We compared our results to those from a large control cohort at the University of Ottawa Heart Institute (UOHI, N = 1582) which had complete cardiovascular phenotyping [16].

Here, we report the results of the first family-based study of the role of CNVs in LS-CHD and identify both cosegregating and *de novo* CNVs enriched in angiogenesis with 25 novel candidate genes that account for up to 10% of disease in our cohort.

## Results

### Cohort description

**Figure 1:**
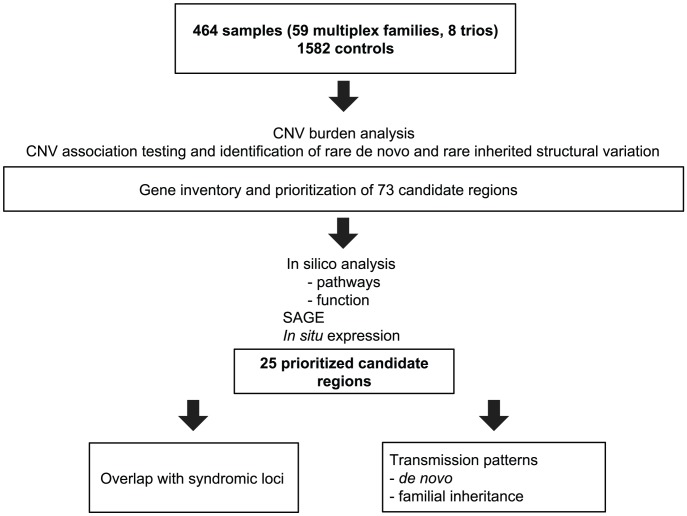
Summary overview of the workflow for CNV detection. Flowchart of sample analysis from recruitment to prioritization.

We accessed a biobank of patients and families with congenital heart disease, centered on the recruitment of French-Canadian multiplex families with LS-CHD [15]. From a total of 464 samples in 67 families genotyped on the Affymetrix 6.0 platform, 174 (37.5%) members were affected with LS-CHD and 290 (62.5%) were confirmed unaffected. A summary by lesion is provided in Table 1 (detailed in Table S1). In 59/67 (83%) families, multiple members were affected with cardiac conditions. In eight families, only one member was affected. In multiplex families, the following relationships between pairs of affecteds were observed: parent-offspring N = 71, sibships/half-sibships N = 30, more distant relationships (grand-parent-grand-child, avuncular, cousins) N = 73. Diagnoses were concordant in 13 multiplex families, discordant in 27, and both concordant and discordant in 17. The median number of affected individuals in multiplex families was two, the maximum nine. A summary overview of the workflow is given in Figure 1.

**Table 1:** Overview over lesions.

Isolated aortic stenosis	19 (10.9)
Isolated aortic root or ascending aorta dilation	20 (11.5)
Isolated BAV	17 (9.7)
Isolated mitral valve defect	13 (7.5)
Isolated CoA	5 (2.8)
More than one LS-CHD lesion	41 (23.6)
LS-CHD lesion and additional CVM	59* (33.9)
Total	174

Distribution of isolated and combined LS-CHD phenotypes. Percents are relative to the total of 174 individuals with cardiovascular malformation.

* This number includes one case with hypoplastic left heart syndrome.

### Measuring the CNV burden and disease association

We compared affected and unaffected individuals with respect to number and size of CNVs, type of CNV (deletion or duplication) and number of genes intersected. Among the LS-CHD cohort, 6,956 autosomal CNVs were detected, amounting to an average of 14.97 autosomal CNVs per individual. We did not detect any statistically significant differences between affected and unaffected individuals in the LS-CHD cohort for overall CNV burden, CNV size, CNV type and number of genes intersected (Table S2).

To search for enrichment of disease associated CNVs within the identified CNVs of the LS-CHD cohort, we first compared affected individuals to unaffected ones using a logistic regression of three different scenarios, which were adjusted for family structure: 1) CNV duplication versus normal CNV state; 2) CNV deletions versus normal CNV state; 3) both CNV duplications or deletions versus normal CNV state. This approach identified 6 enriched genomic loci (pools) of overlapping CNVs (Table S3). After comparison to the well-phenotyped OHI control cohort and public databases, only three pools remained (Figure S4, Table S4), all of which were overlapping segmental duplications.

Next, we evaluated 147 CNVs found to be present only in affected individuals of the LS-CHD cohort. After identical comparisons with controls (Figure S4), 111 unique CNVs were identified which were present only in affecteds. Of the 111 unique CNVs, 73 CNVs remained unique (Table S4 and Figure S5) after accounting for the removal of CNVs based on segmental duplications, 37 as common variants and eight as false negative and positive CNV calls. We found 6/73 of the CNVs to be *de novo* occurrences in the pedigrees, 24/73 were inherited. For the remaining 43/73 CNVs, ancestral information was not available (minimum estimated CNV *de novo* transmission rate of the affected individuals in 53 trios 0.023 and 41 unaffected trios 0.015). Both gains (n = 38) and losses (n = 35) were identified (Table S5).

### Gene inventory and prioritization

In order to describe a role for the genes intersecting within these 73 CNVs in cardiac development, we used PLINK to test for pathway enrichment analysis [17]. Using a rigorous algorithm for pathway enrichment analysis, we found that genes involved in angiogenesis for all identified CNVs, but not other examined gene sets, were significantly enriched in CNVs of affected individuals (genic CNVs p = 0.00867, all CNVs p = 0.0076) (Tables S6 and S7). We next analyzed the gene content of the CNVs by three different approaches in order to evaluate a possible role in cardiovascular biology:

- We conducted *in silico* prediction of gene functionality based on a training set of genes involved in angiogenesis [18]. Our gene set showed three exact matches with the training dataset (*MAPK7*, *ADORA2B* and *ANG*). We identified 26 genes which were significantly enriched (p<0.05) in the LS-CHD cohort (Table S8).
- We used serial analysis of gene expression (SAGE) libraries of embryonic mouse heart libraries to search for genes with at least 3-fold higher expression in the developing outflow tract versus the atria and ventricles at E10.5 [19], [20]. In 8 affected individuals, unique CNVs intersected 16 such genes (Table S9).
- We mined public databases for cardiac-specific function and/or expression patterns of identified genes. Visual inspection of *in situ* expression profiles in the developing mouse identified 19 genes with a strong expression level either in the valves or the heart (Table 2) [20], [21].

**Table 2:** Identified candidate genes.

Gene name	Endeavour	SAGE enrichment	Eurexpress/Genepaint	Transmission pattern	Genomic location
ANG/RNASE4	0.000376	nd	+	Inherited (244, 245)	Chr14q11.2
MAPK7	0.000122	8.15	−	De novo (607)	Chr17p11.2
NCOR1	0.000937	nd	+	De novo (607)	Chr17p11.2
ADORA2B	0.00379	nd	+	De novo (607)	Chr17p11.2
MFAP4	0.00288	11.64	+	De novo (607)	Chr17p11.2
COPS3	0.00379	nd	+	De novo (607)	Chr17p11.2
FLII	0.00187	nd	+	De novo (607)	Chr17p11.2
MSX1	0.0049	7.36	N/A	De novo (599)	Chr4p16
SREBF1	0.00786	nd	+	De novo (607)	Chr17p11.2
SMC1A	0.00906	nd	+	Inherited (17, 20)	ChrXp11.22
LIMS1	0.00496	nd	+	Inherited (389, 390)	Chr2q12
CACNA1C	0.00734	nd	+	n/a(84)	Chr12p13
CRMP1	0.0156	10.96	−	De novo (599)	Chr4p16
RASD1	0.0107	12.83	−	De novo (607)	Chr17p11.2
ERCC5	0.0139	5.65	−	Inherited (92,96)	Chr13q33
ULK2	0.0287	nd	+	De novo (607)	Chr17p11.2
PLA2G12A	0.0409	3.61	−	n/a(717)	Chr10q22
NGEF	0.0454	nd	+	Inherited (106, 107)	Chr2q37
GRPEL1	0.11	3.10	+	de novo (599)	Chr4p16
PRPSAP2	0.34	3.49	+	De novo (607)	Chr17p11.2
MTHFD2	0.84	6.14	+	de novo (834)	Chr2p13
EVC2	0.74	6.19	+	De novo (599)	Chr4p16
CTHRC1	0.17	27.85	+	De novo (243)	Chr8q22
ITGA10	0.00348	nd	+	De novo (390)	Chr1q21
HSD17B10	0.0281	nd	+	Inherited (17, 20)	chrXp11.22

Compilation of the 25 LS-CHD candidate genes fulfilling all selection criteria. From left to right: gene name; ENDEAVOUR *in silico* prioritization; fold overexpression in SAGE experiments outflow tract versus atria/ventricles; *in situ* hybridization in mouse hearts at embryonic day E10.5; transmission pattern (individual IDs); genomic location.

**Figure 2:**
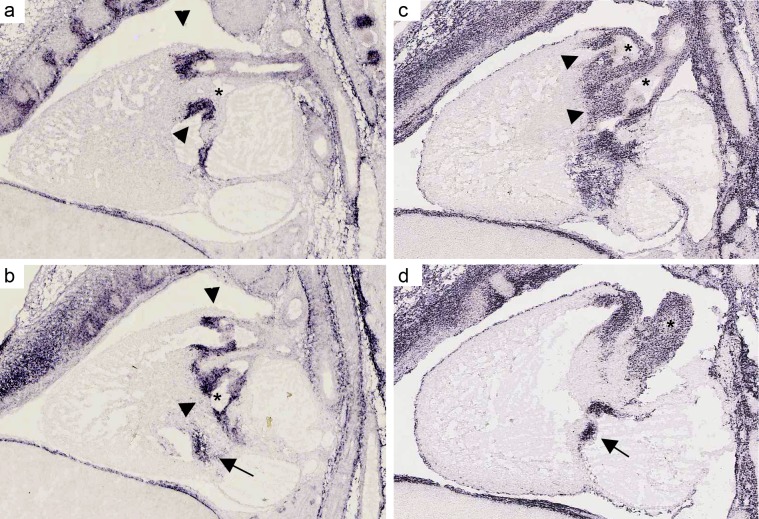
mRNA expression profile of *CTHRC1* and *MFAP4* in embryonic mouse heart. (a, b). *In situ* hybridizations for *MFAP4* of a sagittal section of a wild-type stage E 14.5 mouse heart (c, d) *In situ* hybridizations for *CTHRC1* of a sagittal section of a wild-type stage E 14.5 mouse heart. Both assays show a strong expression in the pulmonary valve (arrows) and aortic/mitral valve (arrowheads). Unlike *CTHRC1* which is more restricted to the valves and only weakly expressed in the endothelium of the aorta, *MFAP4* shows a strong expression in the pulmonary artery and ascending aorta (asterisks). Pictures are taken from Eurexpress (www.eurexpress.org).

Using these three criteria, we identified 25 potential candidates for LS-CHD present in at least two of the three prioritization methods (Table 2). One example is *CTHRC1*, a Wnt cofactor that selectively activates the Wnt/PCP pathway. This gene showed a 28-fold higher expression in the outflow tract versus the cardiac chambers and was specifically expressed within developing valves (Figure 2) [22], [23]. Another example, *MFAP4*, is located within the Smith-Magenis/Potocki-Lupski syndrome region, thought to be an elastin-binding matrix protein involved in cell adhesion and highly expressed in developing valves and great vessels (Figure 2) [24]. Comparing our results with loci suggested by previous studies, we find that only the locus on 10q22 containing *PLA2G12A* overlaps with a locus identified in a linkage scan for hypoplastic left heart syndrome [6]. This gene encodes a secreted phospholipase A(2), is abundantly expressed in the heart and inhibits the BMP-pathway through binding to SMAD-complexes [25].

### Overlap with syndromic loci

Since numerous genetic syndromes are associated with LS-CHD, we searched for overlap between the 25 prioritzed candidates and known loci of such syndromes (Figure 1) [26]. Four regions were thus identified: X-linked Cornelia de Lange (Xp11.22), Ellis-van-Creveld/Witkop/Wolfram syndrome (4p16.1) and Potocki-Lupski syndrome (17p11.2). In addition, we also identified a *de novo* gain at the previously identified 1q21 locus [27].

**Figure 3:**
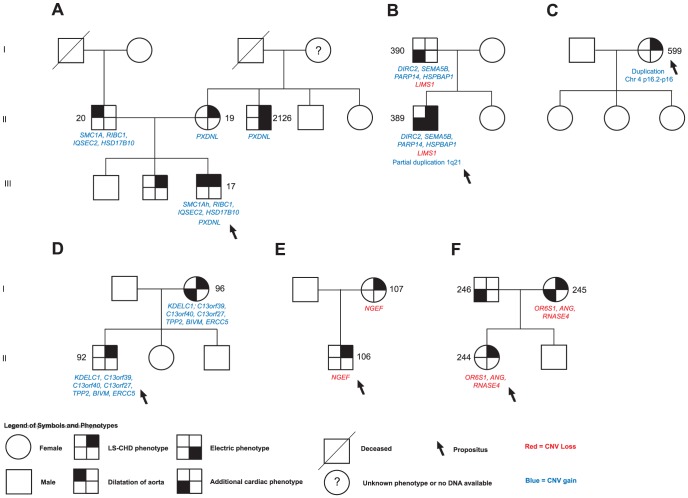
Selected segregation patterns of CNVs in LS-CHD pedigrees. See legend at the bottom of the figure for explanation of symbols. DNA numbers refer to Tables S1, S2, S3, S4, S5, S6, S7, S8, S9 for affected individuals in whom rare CNVs were identified. A.) In family 5, we identified a maternally inherited gain overlapping *PXDNL* and a paternally inherited insertion der(9)ins(X;9)(p11.22;q12) overlapping the Cornelia de Lange syndrome gene SMC1A in the severely affected propositus.(NB: Individual 2126 was not initially genotyped on the Affymetrix 6.0 panel and is therefore not described). B.) The severely affected propositus in family 54 showed three different rare CNVs: a paternally inherited gain overlapping SEMA5B, HSPBAP1, DIRC2 and PARP14, a paternally inherited loss of LIMS1, and a *de novo* partial duplication on chromosome1q21.1. C.) *De novo* occurrence and non-transmission of a large CNV gain (3817 kb) on chr4p16 overlapping the Ellis van Creveld region on chromosome 4. D.), E.) F.) Segregation of prioritized CNVs with disease in families 18, 21 and 39.

**Figure 4:**
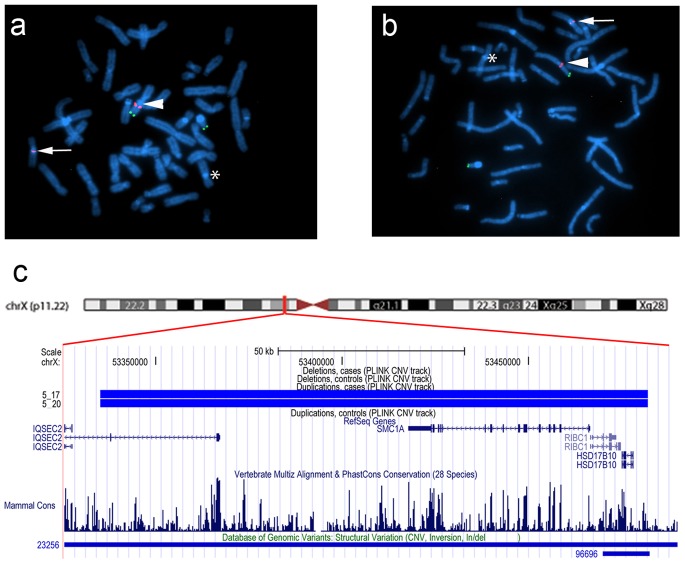
Karyotype der(9)ins(X;9)(p11.22;q12) in family 5. (a,b) FISH was performed on metaphase chromosomes obtained from peripheral blood with a labeled BAC clone that mapped within the detected copy gain (RP11-52N6, red) and a control probe mapped to the Xp/Yp pseudoautosomal region of the sex chromosomes (DXYS129 & DXYS153, green). Green dots show the control probe hybridized to the p arm of chromosomes X and Y. Red dots show the RP11-52N6 BAC clone hybridized on chromosome X (white arrow heads) and in the heterochromatin of chromosome 9 (white arrows). A star shows the normal chromosome 9. These results show that the copy gain is due to a der(9)ins(X;9)(p11.22;q12) in both the father (a) and his son (b). (c). Chromosomal region of the insertion (X;9)(p11.22;q12) in the father and the son of family 5. Four RefSeq genes are identified within this *region IQSEC2, RIBC2, HSD17B10* and the Cornelia de Lange gene *SMC1A*. One larger and one smaller CNV have been detected in the DGV database in this region.

**Figure 5:**
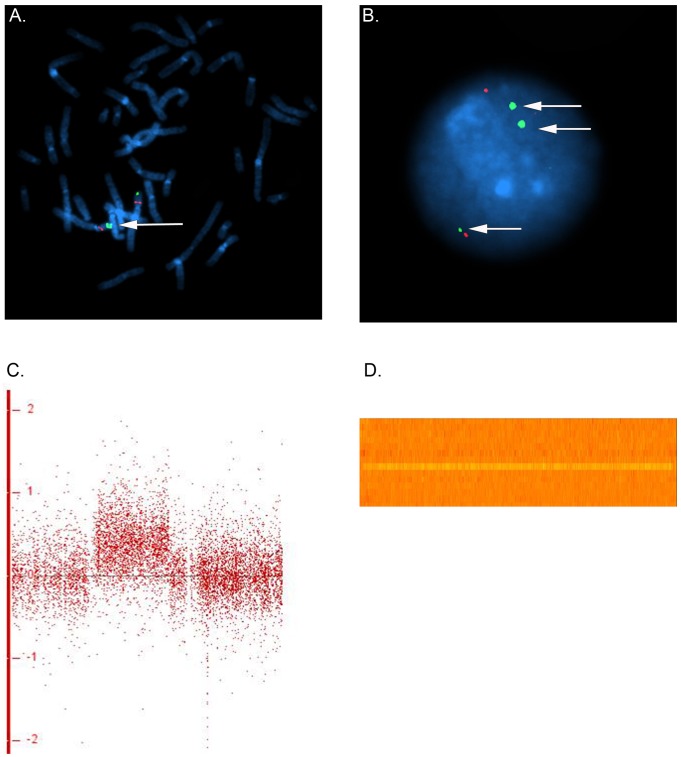
dup(4)(p16.1) in family 43. FISH was performed on metaphase chromosomes and nuclei obtained from peripheral blood with a labeled BAC clone mapped within the detected copy gain (RP11-89K12, green) and a control probe mapped to 4p14 (RP11-332F10, red). (a) Two series of adjacent green dots show the extra copy of the duplicated segment on chromosome 4. (b) The nucleus view with the three green dots showing three copies of the region overlapping the Ellis van Creveld genes on chromosome 4 (c) Log 2 ratio for the large gain in Family 43 on chromosome 4. In general, dots are scattered around 0 along the x-axis for, whereas the identified gain leads to a clear upward shift (d) Heatmap of the identified gain on chromosome 4, each line refers to one individual. An orange row indicates two copies of the region whereas an extra copy leads to a gain in the intensity (yellow line for the individual in family 43).

In family 5, we observed a gain at Xp11.22, encompassing *SMC1A*, *RIBC1*, *IQSEC2* and *HSD17B10*. FISH analysis revealed a 46, ins(X;9)(p11.22;q12) karyotype in the father (Figure 3 and Figure 4). Mutations in *SMC1A* cause the X-linked form of Cornelia de Lange syndrome, in which approximately 25% of patients have CHD, including LS-CHD [28]. In family 43, we detected a duplication on chromosome 4p16.2-16.1 (3817 kb) encompassing 34 genes, including *MSX1*, *EVC* and *EVC2*. The mother was the only individual exhibiting this CNV, all of her 3 children were healthy (Figure 3 and Figure 5). The observed phenotype of aortic valve dysplasia differs from the described cardiac features of Ellis van Creveld syndrome, and no phenotypic overlap with Witkop/orofacial clefting syndromes was apparent [29], [30]. Interestingly, valvular involvement was described in multiple individuals with Wolfram syndrome [30]. In family 8, we detected an affected individual with a 4801 kb gain on chr17p12-p11.2 matching the previously described Potocki-Lupski locus [31]. Cardiac anomalies were present in 26% of the cases with Potocki-Lupski Syndrome, including dilated aortic root, VSD and bicuspid valve, which were all observed in our case [32]. A *de novo* gain at the previously described 1q21.1 locus was found in an individual with BAV, coarctation and ventricular septal defect in family 54 [27]. Taken together, the inventory of the CNV regions overlapping with known syndromic regions reinforces the functional candidacy of the genes identified.

### Transmission patterns

We next sought to determine whether segregation patterns of CNVs containing the most highly prioritized 25 genes would provide additional support for causality (Figure 1). We found that five unique exon-overlapping CNVs segregated with an LS-CHD phenotype in five different families (Figure 3).

In family 5, the ins(X;9)(p11.22;q12) was passed on from the affected father to one affected child, but not to a second affected child. Within this family, the severely affected child received another unique variant from the mother, leading to a gain of the *PXDNL* locus on chromosome 8. This gain was also found in the maternal uncle of the index case known to have BAV. *PXDNL*/*VPO2*, could not undergo the full prioritization workflow, since it is a human-specific gene; high cardiovascular expression has been described for this gene (Figure 3A) [33]. In family 54, we found an inherited loss at the *LIMS1* locus; targeted mouse models of *LIMS1* exhibit cardiovascular phenotypes (Figure 3B) [34]. In addition, this family also shows cosegregation of a rare gain on chromosome 3 (encompassing *PARP14, HSPBAP1, DIRC2, SEMA5*), however, none of the genes contained within this CNV was prioritized in our workflow. In families 18, 21 and 39, a single instance each of vertical transmission of a rare, prioritized CNV was observed (Figure 3D, 3E, 3F). Taken together, 4/5 families with transmission of a prioritized CNV showed discordant phenotypes, only family 21 exhibited concordant phenotypes in both affecteds (Figure 3E). A total of 12 patients in 5 families thus show segregation of a rare, prioritized CNV with LS-CHD. In a further five patients, occurrence of such CNVs was either *de novo* or could not be evaluated further due to lack of ascertainment of ancestors (Table 2). Taken together, CNVs fulfilling all selection criteria were observed in 17/174 affecteds, suggestive of a disease-causing contribution in 10% of our population.

## Discussion

Previous studies have provided evidence for an important role of CNVs in the pathogenesis of several developmental conditions, including congenital heart disease. These studies have predominantly relied on identification of *de novo* CNVs in sporadic cases. Here, we present the first family-based CNV study in LS-CHD, a disorder characterized by familial clustering, reduced penetrance and variable expressivity.

Based on a carefully phenotyped cohort recruited from the French-Canadian founder population and a large number of controls with cardiac evaluation, our findings provide several lines of evidence for a strong association of novel CNVs with LS-CHD. Four plausible syndromic regions and 25 candidate genes either known to be involved in congenital heart pathogenesis or highly likely to impact the risk for LS-CHD were identified.

The use of a family-based cohort allowed us to make use of segregation patterns to strengthen the association between rare CNVs and LS-CHD. In our cohort enriched for multiplex families, CNVs can occur both on an inherited and on a *de novo* basis, mostly with intrafamilial phenotypic variability of LS-CHD. This is compatible with a model in which structural genomic variation contributes to both heritability and variable expressivity of this trait. Interestingly, the vast majority of causative CNVs identified in our study qualify as private in nature, despite our intentional selection bias towards multiplex families within a founder population.

In our studies, we used a sequential filtering approach to increase the biological plausibility of identified LS-CHD candidate genes. Several lines of evidence support enrichment for genes involved in angiogenesis in this disease spectrum. We identified a significant enrichment for genes implicated in angiogenesis, pointing to a role of disturbances in endothelial development in disease pathogenesis. *In silico* analyses, SAGE libraries and mining of public databases identified several known and novel cardiac-specific candidate genes. The *in situ* expression patterns of *CTHRC1* and *MFAP4* are striking examples for enrichment in developing valve structures and endothelium. Interestingly, both of these genes act in known pathways of valvulogenesis and are copy-number gains, suggesting that mechanisms other than haploinsufficiency may contribute to disease pathogenesis in these two examples. Moreover, *CTHRC1* was found to be significantly overexpressed in calcific aortic stenosis, underscoring that hits to developmental genes may predispose to both early and adult onset valve disease [35].

This evidence is further corroborated by the identification of a novel role for known syndromic loci in LS-CHD. Overall, CNVs intersecting with four known syndromic loci were identified, and for all loci, cardiovascular phenotypes were reported. Our study widens the genotype-phenotype correlations in these syndromes; of note, none of the patients had been *a priori* suspected to manifest the associated clinical phenotypes. We suspect that for these loci, the gene dosage – phenotype correlations are not perfect, and that they represent predisposing loci which require further hits for full penetrance of specific clinical features. Taking family 54 as an example, the most severely affected individual showed three unique CNVs, two inherited (one gain, one loss) from the affected father, plus a *de novo* gain overlapping the previously described 1q21 locus (Figure 3). One of the inherited CNVs intersected with *LIMS1*, which plays an essential role in outflow tract development through TGF-β signalling. Interestingly, the clinical phenotypes within this family partially overlapped, strengthening the idea that multiple hits explain reduced penetrance or variable expressivity. Based on this observation, we speculate that other CNVs may also buffer phenotypes; i.e., two antagonistic hits within a single cascade may render cardiac development tolerant against perturbations in an epistatic fashion. Such a model would also be consistent with insight from animal studies in which modifier genes can govern normal or abnormal cardiac development on certain backgrounds [36]. As another example, endothelial-specific knockout of *GATA5* in mice leads to BAV in only 20% of the offspring, compatible with the reduced penetrance even of strong alterations of gene dosage [9]. Other mouse models - examples include mice haploinsufficient for *eNOS*, *Nkx2.5* and *Tbx5* - also display reduced penetrance of CHD traits, with complex gene-dosage effects of interacting alleles [8], [37], [38]. Of note, our study was designed to identify CNVs which would not be detectable by linkage analysis, using an algorithm that prevented the discovery of incompletely penetrant alleles since CNVs seen in unaffected family members or the well-phenotyped control cohort were excluded. Two limitations of our study need to be kept in mind: first, our results do not exclude the possibility that additional, incompletely penetrant CNVs play a role in LS-CHD; second, our design could have missed CNVs containing important non-coding sequences such as regulatory elements since we required further validation through expression studies. Further studies with much larger cohorts are warranted to dispose of sufficient power for the detection of incompletely penetrant alleles, rare double hits and gene deserts [39].

Strengths of our study comprise the stringent, uniform CNV analysis workflow for both the LS-CHD as well as the control cohort, which yielded similar results in respect to reported *de novo* CNV transmission rates [40]. Importantly, we used a rigorous approach limited to CNVs which were unique or statistically enriched in our cases. All controls had adequate cardiac screening to account for mild phenotypes not detectable by conventional clinical examination. Furthermore, the founder character of our cohort theoretically facilitates detection of recurrent hits; nevertheless, this was not the case with our current sample size. On the other hand, several limitations of our study should be noted. At this point, it is unknown whether a cohort enriched for multiplex families with LS-CHD is in itself genetically distinct from a normal population sample. Due to the high stringency of our filtering mechanism, our design precludes the discovery of CNVs with incomplete penetrance and may underestimate the true impact of CNVs on LS-CHD. Furthermore, we recognize that adequate CNV boundary calling remains an issue which will best be resolved using NextGeneration sequencing in future studies.

Taken together, our study suggests that unique CNVs contribute significantly to LS-CHD, and that the majority of genetic events are of private nature. CNVs were found to contribute to 10% of our LS-CHD cases after statistical, biological and genetic validation. Combinatorial interactions between several different genetic factors disturbing key developmental events in left ventricular outflow tract development - such as angiogenesis – may modify the risk for LS-CHD, with important implications for an oligogenic origin for the entire spectrum of LS-CHD.

Future work should aim at more precisely defining gene inventories in larger cohorts and at replication of combinatorial hits in animal models. Insight gained from these studies will assist in identifying the underlying pathophysiological mechanisms of LS-CHD and help clarify the diversity of outcomes in individual patients despite similar morphologies.

## Materials and Methods

### Ethics statement

The ethics committees of Sainte Justine Hospital Research Center, University of Montreal, Centre Hospitalier Universitaire de Québec, Université de Laval, and University of Ottawa approved the study protocol and all participants gave their informed consent. The study was in accordance with the principles of the current version of the declaration of Helsinki.

### LS-CHD cohort

We accessed a biobank of patients and families with congenital heart disease, centered on the recruitment of French-Canadian multiplex families with LS-CHD [15]. A detailed family history (minimum three generations) was obtained from each proband, and all participants provided informed consent. We used a sequential sampling strategy described previously [1]. The cohort was assembled almost exclusively within the French-Canadian population, which is characterized by a marked founder effect and has previously led to the identification of numerous disease genes [14]. We surveyed 464 genomes/individuals in 59 multiplex families and 8 trios of a pedigree-based LS-CHD cohort from Quebec using the Affymetrix Genome-Wide Human SNP Array 6.0 array. The average age was 28 years. A total of 65 French-Canadian families and 2 additional Caucasian families were included in the study. None of the pedigrees had inbreeding or marriage loops. The gender distribution was in favor of males (females N = 223 (48%), males N = 241 (52%)). Index cases with recognizable syndromes, developmental delays and known cytogenetic abnormalities were excluded from the study.

All participants were evaluated by clinical examination, standard 12 lead electrocardiography as well as two-dimensional echocardiography. In 15/464 cases, echocardiography was unavailable. For 6 of these 15 cases, we instead relied on either magnetic resonance imaging, cardiac catheterization or surgical reports to determine phenotype status. For the remaining 9/15 cases, no morphological characterization was available. Standardized two-dimensional and Doppler transthoracic echocardiograms were obtained on all participants through commercially available systems (Hewlett-Packard [Mississauga, Ontario] Sonos 5500, Philips iE33 [Andover, Massachusetts], GE Vivid 7 or Vivid I [Mississauga, Ontario]) according to previously published protocols [1]. Additional anatomic or hemodynamic abnormalities were also recorded. Aortic root dilation was defined as a deviation above a Z score of 2 according to previously published normal values for children or adults [41], [42].

LS-CHD phenotypes were defined as bicuspid aortic valve or other aortic valve disease, coarctation or hypoplastic left heart syndrome. Other cardiovascular phenotypes included dilation of the aortic root/ascending aorta, other cardiovascular malformations, as well as abnormal electrocardiogram/documented arrhythmia. An overview of phenotypes in patients subsequently identified to carry a disease-causing CNV is given in Table S2.

### Control cohort

We accessed genotyping data of a previously described cohort with coronary artery disease and myocardial infarction, the Ottawa Heart Institute cohort, for control purposes [16]. A total of 1582 well-phenotyped controls were used after exclusion of those with LS-CHD, including BAV. Most importantly, subclinical disease, such as asymptomatic bicuspid aortic valve, thus had very little likelihood to escape detection. Moreover, the UOHI cohort was genotyped on the same Affymetrix Genome-Wide Human SNP Array 6.0 platform, with an identical data analysis workflow for CNV detection. The UOHI cohort was matched with respect to gender, but not age. All individuals (cases and controls) in this cohort were used as controls for the detection of rare copy number variants (CNV) and were subjected to the same CNV detection workflow as the LS-CHD cohort. According to 2006 census data, 16% of the population in the Ottawa area, or an estimated 253 individuals in our dataset, are of French-Canadian descent [43].

### Command Console 2.1 and Genotyping Console 3.0.2 quality control

A detailed overview of individual steps in the genotyping and quality control workflow is given in Figure S1. LS-CHD families and control samples were genotyped at the McGill University–Génome Québec Innovation Centre on the Affymetrix Human Genome-Wide SNP Array 6.0. DNA samples from peripheral blood were isolated with standard procedures and master DNA plates were prepared. Following DNA quality determination and sample preparationat the genome facility, cel files were created using AffymetrixGeneChip Command Console software 2.1 and Genotyping Console 3.0.2 (GTC, Affymetrix, Santa Clara, CA, USA) according to the manufacturer's protocol.

### Exclusion of samples for CNV detection QC issues

We used GTC 3.0.2 with a setting of 10 kb and 5 consecutive markers to detect CNVs. We excluded 11 samples that had excessive CNV calls per sample (defined as three standard deviations above the observed mean (49.62 calls per sample, standard deviation 18.14)). The remaining 464 individual samples from 67 families were used for subsequent CNV detection.

### Admixture tests

In order to test for the familiarity within the LS-cohort samples we used Principal Component Analysis (PCA, see Figure S2) [44]. In short, a k-means procedure with 270 samples was used to get the centers of the JPT+CHB, CEU and YRI samples. We projected the first two axes onto the axes running between CEU- JPT+CHB and CEU- YRI centers and formed an oval in the projected space whose major axes were 10 times the length of the standard deviation of CEU cluster along that axis. Samples falling outside the oval were removed. A visual depiction of this process is represented in Figure S3. The returned samples are most likely family derived without a clear European axis. The first and the second component of the PCA were used in the regression analysis to adjust for family structure in the identification of enriched CNV regions.

### CNV identification, validation, and assessment

The analysis was performed using a stringent quality control and copy number detection workflow with a merge procedure relying on two different algorithms for both cohorts (Birdsuite 1.5.5 and GTC 3.0.2) (Figures S1, S4 and S5). Variants meeting the following criteria were retained: 1.) CNVs ≥20 kb, 2.) CNVs either unique or statistically enriched after accounting for relatedness in affected versus unaffected individuals of the LS-CHD and versus the UOHI cohort; 3.) We excluded common CNVs found in the Database of Genomic Variants [45] (DGV Freeze November 2010) 4.) CNVs had to show no more than 50% overlap with known segmental duplications and had to be confirmed by visual inspection. We further prioritized CNVs based on biological plausibility (i.e. expression and pathway analysis) and based on familial segregation with disease (Figure 1). Figures S4 and S5 gives an overview over the workflow used for CNV identification and validation, as outlined in detail below.

### CNV detection workflow and validation

We used a merge procedure of two algorithms to detect CNVs: a) GTC 3.0.2 (Affymetrix) with a setting of minimally 5 consecutive markers/10 kb and b) Birdsuite 1.5.5 (Broad Institute) using default settings (see Birdsuite website for detailed description). Both programs use SNP and copy number probes on the Affymetrix 6.0 array to detect CNVs. CNVs call from GTC 3.0.2 and Birdsuite 1.5.5 were merged using a Python script developed in-house, keeping the outer boundaries for the individual CNV calls. We used a script developed in house to convert Birdsuite's total number of copies on both homologous chromosomes into values for *gains* and *losses* to accurately compare to the output of GTC. For common and known CNPs (results from the Canary algorithm), the mean number of copies (rounded to the closest integer) of each CNP has been computed on all individuals (for the reason that “normal” state of a CNP might not be two in a given population). These integers for each CNP call were then compared to gains and losses called by GTC. For rare or *de novo* CNVs (results from Birdseye), each value has been compared to the “normal” state of two. Finally, we computed the percentage of overlap for each CN segments found between GTC and Birdsuite (with a confidence threshold of 10.0).

Initial validation focused on *de novo* calls of the autosomes by visual inspection of the Heatmap and the log_2_ ratios on GTC 3.02. This showed that the use of 50–100% overlap of the two outputs with a size of >20 kb and a minimum of 5 consecutive probes in the interval was the most reliable method in our hands to detect true CNV calls on the autosomes. In addition, we randomly selected 300 CNV calls from the LS-CHD cohort and examined Heatmap intensities and log_2_ ratios to determine the presence of the CNV. This gave a validation rate of >95%. Therefore we used all CNVs identified in the 50–100% overlapping scenario for subsequent analysis. Sex-chromosomal and autosomal CNVs were analyzed independently (see section below). CNV locations and all genomic coordinates given in this paper are based on the March 2006 Human reference sequence (NCBI build 36.1).

### Analysis of identified autosomal CNVs

Plink 1.07 was used to generate pools of overlapping CNVs (–segment-group). These CNV pools were then tested with SAS 9.2 for statistical evidence of enrichment in affected samples compared to unaffected samples of the LS-CHD cohort. Three different association models within our pools of overlapping CNVs were evaluated: 1) Affected versus unaffected individuals were tested for enriched CNV duplications in comparison to the normal CN state, 2) Similarly for deletions, 3) Similarly for the presence of a duplication or a deletion. We fitted a logistic regression model in SAS 9.2 using *PROC GLIMMIX* conditional on pedigree membership for each CNV using family as a random effect and the number of copies of CNVs as a fixed effect. The following thresholds were used: a p-value less than 0.05 and those significant after Bonferroni correction (9.346E-5 = 0.05/535). A minority of tests did not converge and were tested using a one-sided Fisher exact test.

The identified CNVs enriched in affected individuals along with CNVs found to be uniquely present in affected individuals were then grouped and compared to CNVs from the UOHI cohort. We used Plink 1.07 (–segment-group) to search for overlapping CNVs and tested for enrichment in LS-CHD affected compared to UOHI samples by using a logistic regression model for each CNV adjusted for the first two PCA components to adjust for ethnicity and relatedness. We selected CNVs with a p-value less than 0.05 and those significant after Bonferroni correction (5.56E-3 = 0.05/9).

Plink 1.07 was used to generate a map file (–cnv-make-map). Positions unique to affected individuals of the LS-CHD cohort in the map file were selected and the referring CNV was evaluated with an in house developed Python script for overlap with all identified CNVs of the LS-CHD cohort. Previous studies have highlighted the inaccuracies in determining CNV boundaries using array technologies; these can ideally be addressed in detail by next generation sequencing methods [46], [47]. We therefore examined the boundary calling of inherited CNVs to determine the minimal overlap of seemingly identical CNVs. Identical CNVs based on heatmap calls can vary up to 50% in their overlap when comparing our two-algorithm merge. CNVs overlapping 50% or less with any other CNV in the unaffected individuals of the LS-CHD cohort were regarded as unique compared to the LS-CHD affected individuals. Identified unique CNVs of the LS-CHD cohort were evaluated for 50% overlap with the UOHI-cohort to find CNVs unique to the affected of the LS-CHD cohort and absent from the UOHI cohort. Only CNVs of the affected individuals of the LS-CHD cohort not overlapping with any CNV of the unaffected of the LS-CHD cohort and the UOHI cohort were regarded as unique and were retained for analysis.

### Analysis of sex-chromosomal CNVs

We found a high number of false positive CNV calls (75%) and inaccuracies in calling CNVs on the sex-chromosomes, and opted to visually inspect all CNVs identified in both algorithms in the LS-CHD cohort on heatmaps and log_2_ ratios using GTC 3.0.2. We used Plink 1.07 (–unique) to select CNVs unique to the affected individuals of the LS-CHD cohort and absent from the unaffected of the LS-cohort and the UOHI-cohort.

### Final verification and validation of identified CNVs

Autosomal and sex-chromosomal CNVs found to be uniquely present in LS-CHD affected individuals and autosomal CNVs found to be statistically enriched in LS-CHD affected individuals were considered for further verification (n = 111 unique CNVs and n = 3 enriched CNV regions. We developed a python script to exclude CNVs which were overlapping 50% or more with segmental duplications (UCSC segmental duplications downloaded in January 2011). To account for CNPs and common CNVs we excluded CNVs present with a frequency of more than 0.01% using PLINK 1.07, which roughly corresponds to one CNV in public databases overlapping by 50% or more with our CNVs (DGV database download on November 2010). We examined the position of all identified CNVs in heatmaps and log_2_ ratios, and CNVs with a minimum overlap of 50% in affected family members were regarded as identical CNVs. CNVs present in unaffected family members were removed (n = 8).

We further validated CNV calls made in our *in silico* workflow by using fluorescence *in situ* hybridization (FiSH, for microdeletions>100 kb and duplications >1000 kb) and qPCR. CNV calls were tested in parents and related affected individuals in the same family and more than two independent healthy controls. For a total of 27 calls in 134 individuals, we obtained a confirmation rate of 100% for selected CNVs identified with our strategy (Table S5).

### QPCR validation

The copy number changes identified *in silico* were validated using TaqManGene Copy Number Assays (ABI, Streetsville, ON, Canada) (Table S5). Primers and probes were designed on NCBI genomic sequence (Build36) using the GeneAssist Copy Number Assay Workflow Builder (http://www5.appliedbiosystems.com/tools/cnv/). Each assay was run on quadruplicate samples of genomic DNA. The probe of interest targeting the identified CNV was a FAM dye-based assay; an internal VIC dye-based assay for RNase P was the reference probe. In brief, 10 ng of gDNA, 1xTaqMan probe/primer of the region of interest and 1xTaqMan probe/primer of the internal control in 1xTaqMan Universal Master Mix in a 10 µl reaction was used. The reaction was amplified on the Applied Biosystems7900HT SDS instrument for 2 min at 50°C, 10 min at 95°C, followed by 40 cycles of 15 sec at 95°C and 60 sec at 60°C. Real-time data were collected by the SDS 1.3.2 software. The relative quantification of the test sequence versus the reference gene known to have two copies for autosomal regions was utilized to determine the changes in copy number at the location. Further evaluation of the data and quality checking was done with the SDS 1.3.2 software. Data was then exported as a text file to evaluate for copy number changes in the CopyCaller software according to the manufacturer's guidelines.

### Fluorescence *in situ* hybridization

Metaphase chromosome spreads were prepared from peripheral blood samples following standard cytogenetic protocols. FISH experiments were performed using commercial probes (Cytocell, Cambridge, UK; Abbott Molecular, Des Plaines, IL, USA), or labeled BAC clones from the RP11 library (Centre for Applied Genomics, Hospital for Sick Children, Toronto, ON, Canada), selected according to their mapped position on the Human March 2006 Assembly (hg18) using the University of California at Santa Cruz Genome Browser (Table S5). Slides were pretreated with 2× SSC, dehydrated in ethanol, and left to dry. Chromosomes were denatured in a 70% formamide/2× SSC solution and probes were incubated at 75°C and 37°C before being applied to the slides. Slides were then placed at 37°C overnight for hybridization. Post-hybridization washes were performed in 0.4× SSC, 2× SCC and PBS, and slides were counterstained with DAPI. Chromosomes and probes signals were visualized with a fluorescence microscope (Zeiss, Toronto, ON, Canada) equipped with specific filters. Ten metaphases were scored for each individual, and additional nuclei were examined to confirm duplications. Images were captured and recorded with CytoVision (Genetix, San Jose, CA, USA).

### Gene sets for comparison

Using the key words: ‘Angiogenesis, left ventricular, valve and aortic valve, chondrocyte development and bone development’ gene subsets were downloaded (Table S6) from the Ingenuity application in August 2010. An additional geneset was used from a published list of genes derived from targeted mouse models with cardiac phenotypes [48]. Our list of CNVs intersecting genes from the affected and unaffected individuals was downloaded using a Perl script accessing the Biomart interface at Ensembl (www.ensembl.org).

### Gene enrichment

To test for gene enrichment within the identified CNVs we used an empirical significance test based on a regression framework (-cnv-enrichment-test) implemented in PLINK 1.07. For comparison we downloaded the glist-hg18 from the PLINK website. Enrichment tests were done with respect to all CNVs and all genic CNVs for the above mentioned gene subsets to identify a causal relationship within the total number of identified CNVs in the merge procedure (Table S7) [17].

### Endeavour

In order to prioritize candidate genes for LS-CHD pathogenesis we used the public Endeavour server [18]. The training dataset used was the angiogenesis gene set derived from Inguinity (above), since the gene content of the identified CNVs showed the best match in enrichment testing. We note that the training dataset was significantly enriched in the genes we found to be enriched in the affected versus the unaffected individuals of the LS-CHD cohort. Endeavour generates distinct prioritizations and fuses them into a global ranking using order statistics. We selected the intersecting genes of all our rare CNVs in the affected and unaffected individuals and obtained a prioritization list. Genes passing a threshold of p<0.05 were considered to play a role in disease pathogenesis (Table S8).

### SAGE (Serial Analysis of Gene Expression)

Sage heart libraries were collected from C57BL/6J mice at E 10.5. Related procedures and further analysis for SAGE libraries were described in detail in [20], [49]. Mouse homologs for the human genes identified to interest with the CNVs of the affected and unaffected individuals were obtained from Biomart. To filter for genes with higher expression in the outflow tract (OT), we set a threshold of 3-fold higher tag counts in the OT versus the atria and ventricles. These genes were selected as possible candidate genes for further analysis (Table S9).

### Eurexpress/Genepaint

The presence of two large databases for *in situ* expression in mice enabled us to search for specific expression patterns of candidate genes in endothelium, heart and valves [19], [21]. Using Eurexpress and Genepaint databases, we identified available *in situ* slides of developing mouse embryos at ED 14.5 and visually inspected all available candidate genes. Genes with an elevated expression in the developing heart, valves or vessels were thus identified (Table 2).

## Supporting Information

Figure S1 Flowchart for quality control Affymetrix .cel files.
(EPS)

Figure S2 Principal components analysis (PCA). Principal components were calculated for all unduplicated samples using autosomal SNPs. Comparison of LS-CHD cohort showed a cluster among the CEU samples. The control cohort was also scattered around the CEU samples. With some samples of both cohorts either along the axis of the YRI or JPY+CHB samples.
(TIF)

Figure S3 PCA k-means procedure. We used a k-means procedure to remove outliers. Outlier are marked as red crosses and have not been used in the downstream analysis.
(TIF)

Figure S4 CNV identification workflow. The combination of Birdsuite 1.5.5 and GTC 3.0.2 revealed a total of 7087 CNV calls in the merge procedure of the LS-CHD cohort and 20708 in OHI-cohort.
(EPS)

Figure S5 CNV identification workflow. Hierarchical logistic regression together with principal component analysis(PCA) and selection of unique CNV calls in the affected identified 3 enriched pools of CNVs and 110 autosomal and sex-chromosomal CNVs. 27 CNVs calls have been independently verified either by QPCR or by Fish. 73 Unique calls in the affected of the LS-CHD cohort were identified after removal of overlap with common CNVs and segmental duplications. None of the enriched CNV pools was considered for further analysis, since all three overlapped segmental duplications.
(EPS)

Table S1 Clinical characteristics of all affected individuals in the LS-CHD cohort with a rare CNV. Abnormal echocardiographic or electrocardiographic results are recorded.
(XLSX)

Table S2 CNV burden and de novo transmission rate. The CNV burden (number of autosomal CNVs, number of segments/sample, average segment size, and number of genes spanned by CNV) is given for affecteds and unaffecteds. *De novo* CNVs were determined within all available trios of examined families.
(XLSX)

Table S3 CNV Regions enriched in LS-CHD cohort. A fitted logistic regression model in SAS 9.2 using *PROC GLIMMIX* conditional on pedigree membership for each CNV with family as a random effect and the number of copies of CNVs as a fixed effect was used. P-values less than 0.05 and those significant after Bonferroni correction were taken.
(XLSX)

Table S4 CNV Regions enriched after adjusting for family structure and comparison with OHI cohort. The first and the second component of the PCA were used in the regression analysis to adjust for family structure in the identification of enriched CNV regions.
(XLSX)

Table S5 Full list of unique inherited and de novo CNV identified in affected individuals with LS-CHD using stringent selection criteria.
(XLSX)

Table S6 LS-CHD related gene subsets for enrichment test. Gene subsets for key processes involved in LS-CDH were downloaded from Ingenuity.
(XLSX)

Table S7 Pathway analysis. An empirical significance test based on a regression framework was used for enrichment testing of the LS-CHD pathway genes relative to all and all genic CNVs.
(XLSX)

Table S8 Endeavour prioritization list for the LS-CHD cohort. The enriched angiogenesis dataset was used to prioritize candidate genes for LS-CHD pathogenesis to generate a global ranking using order statistics.
(XLSX)

Table S9 SAGE analysis for genes identified in the LS-CHD cohort. The mouse homologues for the human genes intersecting rare CNVs were filter for enriched expression in the outflow tract versus ventricle and atrium in developing mouse hearts.
(XLSX)
