# Correction: Rare Copy Number Variants Contribute to Congenital Left-Sided Heart Disease

**DOI:** 10.1371/annotation/8bc63544-9ed4-42ca-a830-e8058ab13bab

**Published:** 2013-03-28

**Authors:** Marc-Phillip Hitz, Louis-Philippe Lemieux-Perreault, Christian Marshall, Yassamin Feroz-Zada, Robbie Davies, Shi Wei Yang, Anath Christopher Lionel, Guylaine D'Amours, Emmanuelle Lemyre, Rebecca Cullum, Jean-Luc Bigras, Maryse Thibeault, Philippe Chetaille, Alexandre Montpetit, Paul Khairy, Bert Overduin, Sabine Klaassen, Pamela Hoodless, Philip Awadalla, Julie Hussin, Youssef Idaghdour, Mona Nemer, Alexandre F. R. Stewart, Cornelius Boerkoel, Stephen W. Scherer, Andrea Richter, Marie-Pierre Dubé, Gregor Andelfinger

Philip Awadalla, Julie Hussin, and Youssef Idaghdour should be included in the author byline. Philip Awadalla should be listed as the 19th author and contributed materials/analysis tools, Julie Hussin should be listed as the 20th author and contributed by analysing the data, and Youssef Idaghdour should be listed as the 21st author and contributed reagents/materials/analysis tools. The affiliation of these three authors is 1: Cardiovascular Genetics, Department of Pediatrics, Centre Hospitalier Universitaire Sainte Justine, Université de Montréal, Montréal, Québec, Canada.

