# Supplementary figures and images for: Rare Copy Number Variants Contribute to Congenital Left-Sided Heart Disease
Source: PLoS Genet. 2012 Sep 6;8(9):e1002903. doi: 10.1371/journal.pgen.1002903 (PMC3435243; doi:10.1371/journal.pgen.1002903)

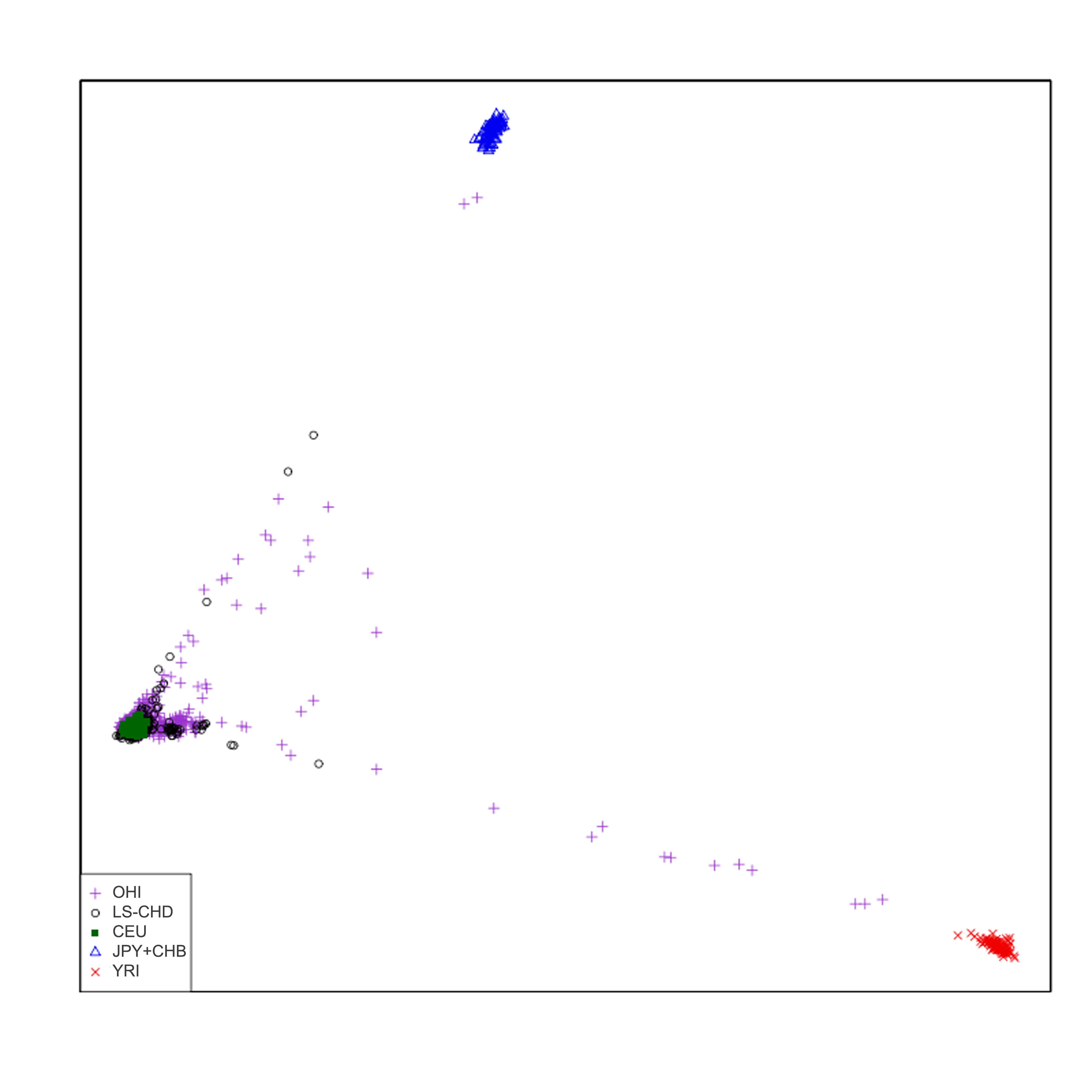

Supplement: Figure S2 — Principal components analysis (PCA). Principal components were calculated for all unduplicated samples using autosomal SNPs. Comparison of LS-CHD cohort showed a cluster among the CEU samples. The control cohort was also scattered around the CEU samples. With some samples of both cohorts either along the axis of the YRI or JPY+CHB samples. (TIF) [file pgen.1002903.s002.tif]

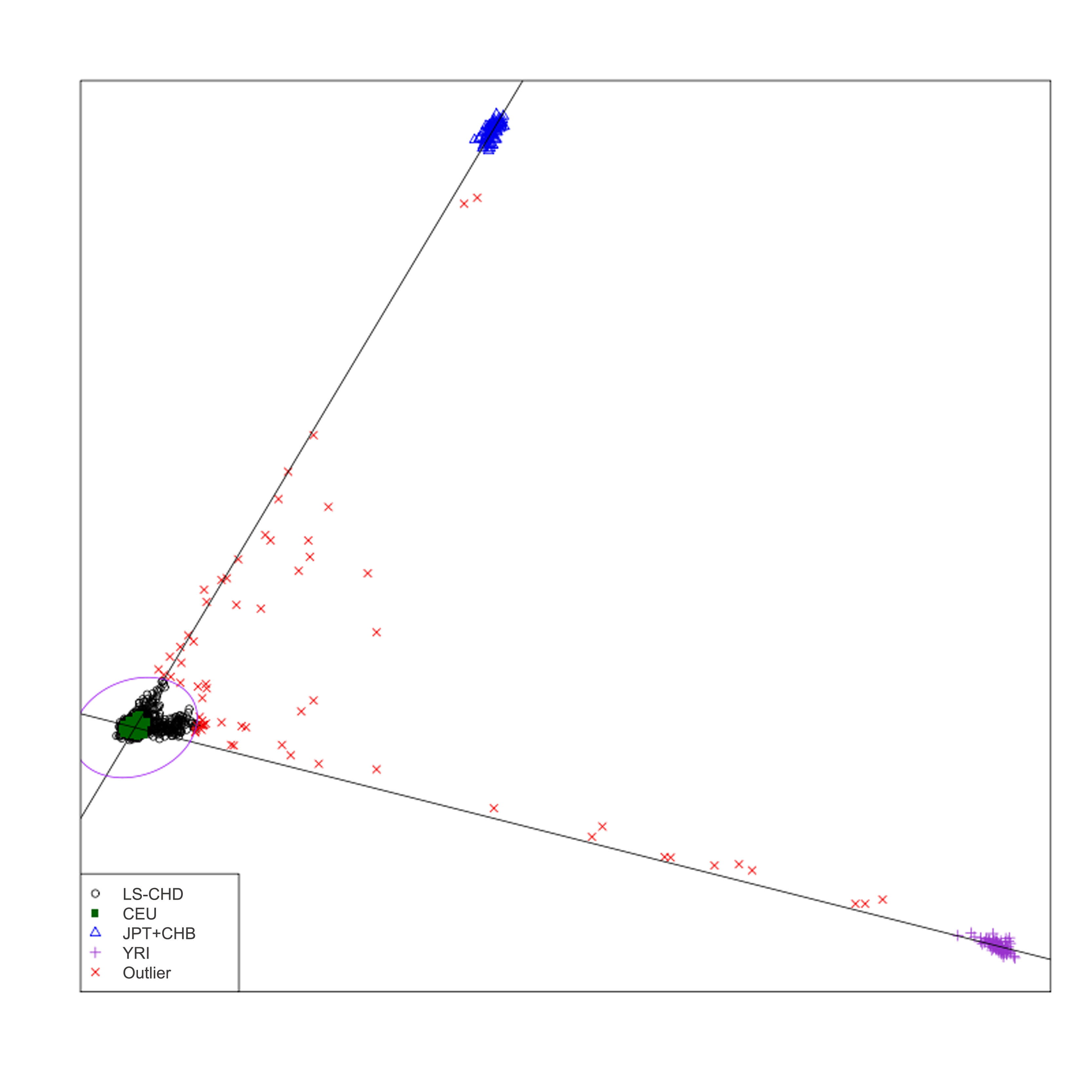

Supplement: Figure S3 — PCA k-means procedure. We used a k-means procedure to remove outliers. Outlier are marked as red crosses and have not been used in the downstream analysis. (TIF) [file pgen.1002903.s003.tif]
